# Effect of Sulforaphane and 5-Aza-2’-Deoxycytidine on Melanoma Cell Growth

**DOI:** 10.3390/medicines6030071

**Published:** 2019-06-27

**Authors:** Tung-chin Chiang, Brian Koss, L. Joseph Su, Charity L. Washam, Stephanie D. Byrum, Aaron Storey, Alan J. Tackett

**Affiliations:** 1 Department of Environmental and Occupational Health, University of Arkansas for Medical Sciences, Little Rock, AR 72205, USA; 2 Department of Biochemistry & Molecular Biology, University of Arkansas for Medical Sciences, Little Rock, AR 72205, USA; 3 Winthrop P. Rockefeller Cancer Institute, Cancer Prevention and Population Sciences Program & Department of Epidemiology, University of Arkansas for Medical Sciences, Little Rock, AR 72205, USA; 4 Arkansas Children’s Research Institute, Little Rock, AR 72202, USA

**Keywords:** sulforaphane, epigenetic, 5-aza-2’-deoxycytine, melanoma

## Abstract

**Background:** UV exposure-induced oxidative stress is implicated as a driving mechanism for melanoma. Increased oxidative stress results in DNA damage and epigenetic dysregulation. Accordingly, we explored whether a low dose of the antioxidant sulforaphane (SFN) in combination with the epigenetic drug 5-aza-2’-deoxycytidine (DAC) could slow melanoma cell growth. SFN is a natural bioactivated product of the cruciferous family, while DAC is a DNA methyltransferase inhibitor. **Methods:** Melanoma cell growth characteristics, gene transcription profiles, and histone epigenetic modifications were measured after single and combination treatments with SFN and DAC. **Results:** We detected melanoma cell growth inhibition and specific changes in gene expression profiles upon combinational treatments with SFN and DAC, while no significant alterations in histone epigenetic modifications were observed. Dysregulated gene transcription of a key immunoregulator cytokine—C-C motif ligand 5 (CCL-5)—was validated. **Conclusions:** These results indicate a potential combinatorial effect of a dietary antioxidant and an FDA-approved epigenetic drug in controlling melanoma cell growth.

## 1. Introduction

While the incidence of certain cancer types has declined, the number of diagnosed melanoma cases has increased sharply over the past three decades [1,2]. Ultraviolet (UV) exposure is one of the most apparent risk factors for melanoma [3]. There are many types of photosensitizers, such as DNA, melanin, and tryptophan, that can receive UV energy and result in direct DNA damage and ROS accumulation [4,5,6]. UVB affects DNA by forming cyclobutane pyrimidine dimers (CPDs), which lead to DNA mutation [7]. UVA directly induces oxidative stress through the accumulation of 8-oxo-7,8-dihydroguanine (8-oxo-G) and other photoproducts [4]. UV also induces melanin synthesis [8]. There are two types of melanin, eumelanin, and pheomelanin. The ratio of the two types of melanin is dependent on the polymorphism of the melanocortin-1 receptor (MC1R) gene and results in differential pigmentation [6,9]. Synthesis of eumelanin leads to scavenges of reactive oxygen species (ROS) while the synthesis of pheomelanin leads to depletion of antioxidants and results in ROS accumulation [10,11,12]. This is in concert with the determination that people with pale skin and red hair have low eumelanin and high pheomelanin and are known to have a higher risk of melanoma [13]. Many studies have identified another photosensitizer, tryptophan, which utilizes the energy from UVA and UVB to form a tryptophan photoproduct, 6-formylindolo(3,2-b) carbazole (FICZ) [14,15]. FICZ has a high affinity to the aryl hydrocarbon receptor (AhR) and activates AhR response genes, including cyclooxygenase-2 (Cox2), a melanoma prognostic marker gene [16], and cytochrome P4501A1, which increases ROS accumulation [17]. It has been shown that UVB activates AhR responses that decrease the tumor suppressor gene p27 and impairs nucleotide excision repair (NER) resulting in DNA mutation [18]. In addition to UV exposure, other environmental factors such as cigarette smoking, environmental dioxin 2,3,7,8-tetrachlorodibenzo-p-dioxin (TCDD), and arsenic exposure also induce melanogenesis [3,19,20,21,22]. Accumulated ROS from UVA and UVB via different photosensitizers, as well as environmental exposures, have many impacts on cell growth and defense. These impacts include inhibition of p27, cell cycle regulation, increased cytokines, decreased antioxidant glutathione s-transferase, increased 8-oxoG, activation of mitogen-activated protein kinase/extracellular signal-regulated kinases1/2 (MAPK/ERK1/2), increased cell proliferation and decreased tumor suppressor gene p16 [5]. These findings support the fact that melanoma patients have a higher level of oxidative stress and that this stress is associated with the progression of the disease [23].

Studies have shown that environmental exposure-induced DNA damage and oxidative stress can also result in epigenetic changes [24,25,26,27]. Elevated ROS is associated with DNA methylation and histone post-translational modifications (PTMs) [25,26,27]. DNA hypermethylation at promoter CpG sites, especially at tumor suppressor gene promoters, is associated with silencing gene expression in a variety of cancers, including melanoma [28,29]. Many tumor suppressor genes related to cell cycle progression, DNA repair, and apoptosis are methylated in different stages of melanoma [5,24,30,31,32,33]. Whole genome DNA methylation profiles from advanced melanoma patients have uncovered a differential methylation pattern that is correlated with survival rates [34]. In addition to aberrant DNA methylation, histone PTMs play critical roles in cancer development independently, in combination with other histone PTMs, and interactively with DNA methylation [24]. Our lab identified the elevation of trimethylation of lysine 27 on histone H3 (H3K27me3) in metastatic melanoma relative to primary melanoma [35]. H3K27me3 is catalyzed by the protein Enhancer of Zeste 2 (EZH2), a member of the Polycomb-group (PcG) family. EZH2 can recruit DNA methyltransferase (DNMT1) to chromatin to form a multisubunit protein complex that suppresses gene expression [36]. 

Epigenetic therapy using 5-aza-2’-deoxycytidine (DAC), an FDA-approved DNA demethylation agent, has been successfully used to treat myelodysplastic syndromes (MDSs) either alone or in combination with other drugs [37,38,39]. DAC is a deoxycytidine analog with the replacement of nitrogen at position 5 of the pyrimidine ring [40]. DAC interferes with normal DNA methylation by forming an irreversible covalent bond with DNMT1 [41]. The subsequent DNA-DNMT adducts play a role in controlling cancer cells depending on the dose of DAC. At high doses, DAC induces cytotoxicity by accumulated DNA–DNMT1 adduct-induced apoptosis and DNA synthesis arrest. At low doses, DNA synthesis is continued, while DNA–DNMT1 adduct bonds are being degraded and repaired, resulting in systematically hypomethylated DNA [41,42]. Studies show that DAC has effects on melanoma via decreasing cell growth and invasion [43] as well as alerting gene expression, includes tumor suppressor genes [44]. 

Regulating oxidative stress via the consumption of antioxidant-rich cruciferous vegetables (e.g., broccoli and Brussels sprouts) has been well-studied in cancer prevention [45,46,47]. One of the common compounds from cruciferous vegetables with cancer prevention characteristics is glucosinolate. Glucosinolate is not bioactivated until the enzyme myrosinase is released from the plant cell, by chewing or through denaturing by cooking, to catalyze a hydrolytic reaction to form isothiocyanates (ITCs) [48]. Sulforaphane (SFN) is one of the promising anticancer ITCs and can induce biphasic biological impact via generating different level of ROS depending on their doses [48,49]. At a dietary dose, SFN-derived ROS stimulate antioxidant protein expression to balance the ROS level induced from UV exposure. SFN activates nuclear erythroid 2-related factor 2 (Nrf2) to bind to the antioxidant response element at the promoter region of Nrf2-regulated genes. Those genes are phase-II detoxification enzymes (e.g., glutathione S-transferase, quinone reductase, and glucuronosyltransferase). By doing this, SFN increases antioxidant capacity. Furthermore, phase-I enzymes such as P450s, which activate toxic chemical compounds, are reduced by SFN at dietary doses [50,51,52]. In this way, SFN delivers chemopreventive effects through strengthening cell defense systems by increasing antioxidant enzymes and reducing carcinogen toxicity. Studies have shown that SFN acts as a cell-killing agent at high doses. At a high concentration of SFN, elevated amounts of SFN-derived ROS accumulate in the cells, mitochondrial function is disrupted [49], cell proliferation is blocked, cell cycle G2/M is arrested, and caspase-mediated apoptosis is induced [48,52,53,54,55]. High concentrations of SFN also induce epigenetic modification. Studies show that high doses of SFN enhance global histone acetylation by inhibiting histone deacetylase (HDAC) activity and reducing cell growth in prostate cancer [56,57]. The dual roles of SFN in cytoprotection and slowed tumor growth, as well as the low toxicity, are cell-specific [48]. Where and how the ROS is formed by SFN and the impact of surrounding molecular environments has gained great interest in research either with SFN alone or in combination with other chemotherapy drugs in many cancers [58,59,60]. 

The research reported here seeks to determine whether combining DAC and SFN can synergistically slow melanoma cell growth. We aimed to utilize a dietary dose of SFN as a natural antioxidant, while at the same time suppressing gene transcription with a low dose of the clinically approved epigenetic modifier DAC. We rationalized that with lower oxidative stress, the low dose of DAC could deliver its epigenetic effect without inducing cytotoxicity. This study is the first step in testing the combined effect of DAC and SFN in a mouse melanoma cell line. Cell growth characteristics, gene expression profiles, and histone PTMs are compared between single and combination treatments of DAC and SFN using mouse melanoma cells. Our data show cell growth inhibition, dysregulation of gene transcription, and increased cytokine production with combination treatment compared to individual treatments. Histone PTMs were identified but did not show differences following treatment. This in-vitro data provides a path to investigate the role of target gene sets and the potential role of the dietary antioxidant SFN in melanoma treatment and prevention.

## 2. Materials and Methods 

### 2.1. Cell Culture and Treatment 

Mouse melanoma B16F10 cells were obtained from ATCC and maintained in Dulbecco’s Modified Eagle Medium (DMEM) (ThermoFisher, Waltham, MA, USA) supplemented with 10% FBS (ThermoFisher, Waltham, MA, USA) and 1% penicillin/streptomycin (ThermoFisher, Waltham, MA, USA). Cells were checked for mycoplasma contamination by MycoAler PLUS Mycoplasma Detection Kit (Lonza Walkersville, Walkersville, MD, USA) before experiments. 

IC50s for both drugs were determined by using CellTiter 96 AQ_ueous_ One Solution Cell Proliferation Kit (Promega, Madison, WI, USA), following the manufacturer’s protocols. In brief, cells were seeded at 1500 cells/well in a 96-well plate for 24 h. Cells were then treated with 5-aza-2′-deoxycytidine (DAC) (Sigma Aldrich, St. Louis, MO, USA) dissolved in dimethyl sulfoxide (DMSO) at concentrations ranging from 25 µM to 6.1 nM (4-fold dilutions from 25 µM, 6.25 µM, 1.56 µM, 390 nM, 97.7 nM, 24.4 nM, to 6.1 nM) for 72 h; and sulforaphane (LKT labs, St Paul, MN, USA) dissolved in water at concentrations ranging from 352 µM to 86 nM (4-fold dilutions ranging from 352 µM, 88.1 µM, 22 µM, 5.5 µM, 1.37 µM, 344 nM, to 86 nM) for 48 h. Dimethyl sulfoxide (DMSO) (Sigma Aldrich, St. Louis, MO, USA) was used as a control in the DMSO wells, at 0.00025%, equivalent to the highest amount of DMSO in the highest dose of treatment (10 mM DAC in DMSO was freshly diluted 400,000 times to 25 nM in culture medium). 

Preliminary tests with different doses and duration were performed, based on the results from IC50 measurements, in 6-well plates. The optimal doses and duration of treatments were chosen based on the number of viable cells with greater than 50% of cell survival at single treatment for DAC and SFN, with fewer cells surviving with combination treatment. SFN at 5 µM and DAC 25 nM were determined to be an optimal dose in the preliminary tests. Cells were seeded in 6-well plates at 4 × 10^4^ cells/well and were allowed to attach for 24 h. For combinatorial drug treatment, cells were treated with DAC at 25 nM for 24 h, the medium was removed, and fresh medium with 25 nM DAC and 5 µM SFN was added. Cells were then incubated for another 48 h. For DAC or SFN single treatment, cells were treated with only DAC or SFN following the same operations as a combination treatment. All treatment groups were harvested at the same time for different target analysis, which included cell number counting and measurements of apoptosis, cell cycle, and gene transcription. Three independent biological repeats were performed.

For cytokine analysis, cells were seeded in 10 cm dishes at 3 × 10^5^ cells/dish and were treated with SFN and DAC as described above in 10% serum-containing medium. Culture medium was replaced from 10% to 1% serum-containing medium with the same dosing scheme at the last 24 h of treatment. The purpose is to reduce potential background. Also, the final culture medium was reduced from 10 mL to 5 mL to increase the concentration of cytokine in the supernatant. The supernatant of each dish was collected for cytokines array analysis. The cell number is calculated to adjust the final amount of supernatant to be loaded from even amount of cells for cytokine analysis.

For CCL5 enzyme-linked immunosorbent assay (ELISA) analysis, cells were grown and treated as described for cytokine array analysis, except the initial cell density is at 2 × 10^5^ cells per10 cm dish, and the final culture medium was reduced from 10 mL to 5 mL. 

For histone analysis, cells were seeded in 10 cm dishes at 2 × 10^5^ cells/dish and were treated with SFN and DAC as described above. Additionally, EZH2 inhibitor EPZ6438 (Med Chem Express, Monmouth Junction, NJ, USA), was used at 5 µM to treat cells for 48 h for analysis of histone epigenetic post-translational modifications. The dose of EPZ6438 was selected for optimal inhibition of the catalytic output of EZH2, histone H3K27me3, and was used as a positive control for histone analysis. DMSO at 0.05%, equivalent to the highest amount of DMSO in the treatment (10 mM EPZ6438 in DMSO was freshly diluted 2000 time to 5 µM in culture medium) was used in the control plates. Three independent biological repeats were performed. 

### 2.2. Assays for Characteristics of Cell Growth 

#### 2.2.1. Viable Cell Count 

Cell number was counted with Trypan blue solution (0.4%) using a hemocytometer. The number of the cell count was controlled to within 20–50 cells/square via dilution of cells before mixing with trypan blue. 

#### 2.2.2. Cell Cycle Arrest Analysis

Cells cycle was analyzed by fixing cells in 70% ethanol overnight and staining with propidium iodide (PI)/RNase Staining Buffer (BD Biosciences, San Jose, CA, USA). The stained DNA was analyzed at the University of Arkansas for Medical Sciences (UAMS) flow cytometry core with an LSRFortessa Flow cytometer (BD Biosciences, San Jose, CA, USA). Flow cytometry data were analyzed with Flow Jo (Ashland, OR, USA) and Dean-Jett Fox (DJF) model (BD, Franklin Lakes, NJ, USA).

#### 2.2.3. Apoptotic Analysis 

Apoptosis was measured by Annexin V and 4′,6-diamidino-2-phenylindole (DAPI) staining using the annexin V-FITC apoptosis detection kit (BD Pharmigen, San Jose, CA, USA), following the manufacturer’s protocol. Cells were analyzed at the UAMS flow cytometry core with an LSRFortessa Flow cytometer (BD Biosciences, San Jose, CA, USA). Flow cytometry data were analyzed with Flow Jo (Ashland, OR, USA).

### 2.3. RNA-Seq Analysis

#### 2.3.1. RNA Extraction and Targeted Gene Expression Analysis 

RNA was extracted with the RNeasy Mini Kit (Qiagen, Germantown, MD, USA) following the manufacturer’s protocols and eluted in water. RNA was reversed transcribed into cDNA with the One Step iScript kit (BioRad, Hercules, CA, USA) following the manufacturer’s protocol.

Targeted genes of interest were amplified with 20 ng of cDNA, SYBR green Supermix (Bio-Rad, Hercules, CA, USA) and primers (final concentration at 750 nM). The PCR cyclic conditions used were 95 °C for 3 min, followed by 39 cycles of 98 °C for 15 s and 57 °C for 30 s. The following primer pairs (Integrated DNA Technologies, Coralville, IA, USA) were used for real-time analysis (Table 1): 

#### 2.3.2. RNA-Seq Sample Preparation

cDNA libraries were constructed using Illumina’s TruSeq stranded mRNA sample preparation kit according to the manufacturer’s protocol. Briefly, 500 ng of total RNA was polyA selected, chemically fragmented, and converted to single-stranded cDNA using random hexamer-primed reverse transcription. Second strand synthesis was then performed to generate double-stranded cDNA, followed by fragment end repair and the addition of a single A base to each end of the cDNA. Adapters, including a 3’ adapter and a 5’ adapter containing 1 of 48 unique indexes, were then ligated to the fragment ends to enable attachment to the sequencing flow cell and sample pooling. Next, library DNA was PCR amplified and validated for fragment size and quantity using an Advanced Analytical Fragment Analyzer (AATI) and Qubit fluorometer (Life Technologies), respectively. Equal amounts (5 µL of 4 nM dilutions) of each library were pooled and 5 µL of the pool was denatured for 5 min by the addition of 5 µL of 0.2 N NaOH, incubated at room temperature for 5 min, neutralized by the addition of 5 µL 200 mM Tris pH 7.0, and diluted to a loading concentration of 1.8 pM; 1.3 mL of the denatured, diluted library was added to a NextSeq reagent cartridge V2.0 for sequencing on a NextSeq 500 platform using a high output flow cell to generate approximately 25 million 75-base reads per sample. All sequencing was conducted by the Center for Translational Pediatric Research Genomics Core Lab at Arkansas Children’s Research Institute (Little Rock, AR, USA).

#### 2.3.3. RNA-Seq Data Analysis

RNA reads were checked for quality of sequencing using FastQC v.0.11.7 (http://www.bioinformatics.babraham.ac.uk/projects/fastqc/). The adaptors and low-quality bases (Q < 20) were trimmed to a minimum of 36 base pairs using Trimmomatic v0.38 [61]. Reads that passed quality control were aligned to the mouse (mm10) (GCA_000001305.2) reference genome using TopHat v2.1.1 [62]. Sample alignment files (.bam) were then imported into Blast2GO v5.1.13, and gene level expression counts quantified using htseq [63,64]. Only reads uniquely aligned to known genes were retained and counted. Multimapped reads were discarded. Genes with low counts were then removed before downstream analysis. To retain the maximum number of interesting features genes with a minimum of 1 counts-per-million (CPM) values in at least 3 libraries were retained for further investigation. The filtered dataset was then normalized for compositional bias using a trimmed mean of M values (TMM) and log_2_ transformed [65]. For each comparison, edgeR quasi-likelihood method (glmQLFTest) correcting for batch effect was used to identify differentially expressed genes between experimental groups [2]. Genes with multiple tests corrected (FDR) *p*-values of 0.05 [66] and a fold change > 2 were selected for further comparisons between treatments and analyzed by Ingenuity Pathway Analysis (IPA) for biological involvement. 

### 2.4. Chemokines Analysis 

The supernatant of control and combination treated groups was spun at 10,000 g for 5 min to remove the cell debris. The supernatant was added to the membrane of Proteome Profiler mouse XL Cytokine array kit (R&D system Inc, Minneapolis, MN, USA). The manufactural protocol was followed with modification at the final film developing. Western Lightning Plus-ECL (PerkinElmer, Waltham, MA, USA) was applied at the end of film developing to have clear signals. 

For ELISA, the supernatant was spun at 10,000 g for 5 min to remove the cell debris and further diluted 10 times in 1 × PBS. Duplicate diluted supernatant from each group and the serially diluted standards (ranging from 7.8 pg/mL to 500 pg/mL) were tested for the level of CCL5 according to the manufacturer’s instructions (R&D Systems Inc., Minneapolis, MN, USA). The cell number is also calculated and applied in data analysis to reflect the level of CCL5 in the supernatant is from the same amount of cells.

### 2.5. Histone PTM Mass Spectrometry 

Histones were purified from approximately 5 million cells by acid extraction, as described by Taverna, SD et al. [67]. The amount of protein was quantified by BCA Protein Assay Kit (ThermoFisher, Waltham, MA, USA). Extracted histones (5 µg), were resolved on a 4–20% gradient SDS-PAGE gel. Histone bands were visualized by staining with GelCode Blue (Thermo). Histones were excised from the gel, destained, and treated with 20 µL/band of 30% d6-acetic anhydride in 50 mM ammonium bicarbonate. Histones were then digested in-gel with 125 ng/band sequencing-grade trypsin at 37 °C overnight. Acidified tryptic peptides were separated using a 2.5 µm Waters XSelect CSH resin on a 150 mm × 0.075 mm column using a nanoAcquity UPLC system (Waters, Milford, MA, USA). Peptides were separated using a 60-min chromatography gradient, with a 40-min linear separation gradient from 97% buffer A (0.1% formic acid in water (v/v)), 3% buffer B (0.1% formic acid (v/v), 99.9% acetonitrile (v/v)), to 80% of buffer A, 20% buffer B. Eluted peptides were ionized by electrospray (2150 V) and analyzed on an Orbitrap Fusion Lumos mass spectrometer (Thermo Fisher, Waltham, MA, USA) using data-dependent acquisition. A full-scan MS was acquired in profile mode at 120,000 resolution from 375 to 1500 m/z (AGC target 5 × 10^5^, max injection time 100 ms), followed by data-dependent MS/MS analysis with a 3 second duty cycle time. Peptides with a determined monoisotopic peak, intensity threshold greater than 2 × 10^4^ counts, and charge state of 2–7 were selected for HCD fragmentation at 30% collision energy, AGC target of 1 × 10^4^, maximum injection time 35 milliseconds, and analyzed in the ion trap with scan speed set to rapid.

Raw data files were analyzed using Mascot (Matrix Science, London, UK) using a custom Uniprot database which included only mouse histones (Table 2). Files were searched with a precursor tolerance of 3 ppm and fragment ion tolerance of 0.5 Da. Fixed modifications included carbamidomethylation of cysteine. Variable modifications to lysine included monomethylation, dimethylation, trimethylation, acetylation, deuterated acetylation, and methylation and deuterated acetylation. Variable modifications to arginine included monomethylation and dimethylation. Variable modifications to serine and threonine were phosphorylation. Up to four missed trypsin cleavages were permitted. Mascot search results were loaded into Scaffold, and filtered for a protein FDR of 1%, a peptide score probability of 80%, and a minimum of 5 peptides per protein. Spectral count data was exported in tabular format and analyzed using R [68]. 

## 3. Results

### 3.1. SFN and DAC Single and Combination Treatment Result in Growth Inhibition 

**Figure 1:**
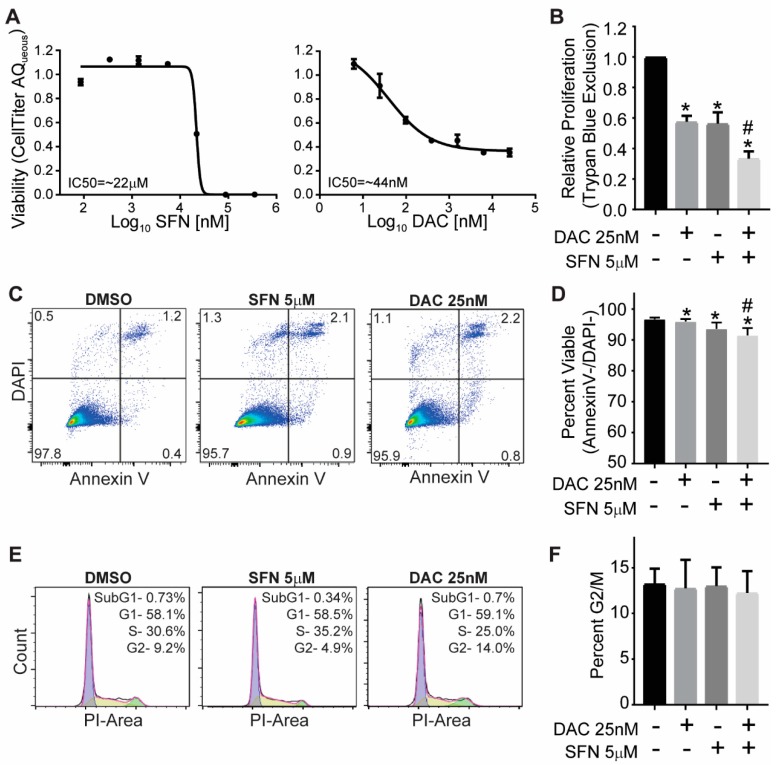
Impact of SFN and DAC single and combination treatment on the growth of B16 melanoma. (**A**) The IC50 of SFN and DAC single treatment is approximately 22 µM and 44 nM, respectively. Cell viability was determined using the CellTiter 96 AQ_ueous_ One Solution Cell Proliferation kit. The data were analyzed by nonlinear regression to determine the IC50. (**B**) Growth inhibition induced from single and combination treatment of SFN and DAC. Viable cells were measured by trypan blue staining and analyzed by Student’s *t*-test. (**C**) Representative apoptosis analysis (AnnexinV/DAPI) by flow cytometry from control, SFN, and DAC single treatment. (**D**) The percentage of viable cells with DAC and SFN single and combination treatments were compared to control. (**E**) Representative cell cycle analysis from control and SFN and DAC single treatment. Data were analyzed with Flow Jo /Dean-Jett Fox (DJF) model. (**F**) The percent G2/M phase in DAC and SFN single and combination treatments were compared to control with Student’s *t*-test. * Significantly different from control, # Single treatment is significantly different from combinational treatment (Student’s *t*-test).

The IC50 for SFN was calculated to be approximately 22 µM for SFN and 44 nM for DAC (Figure 1A). For cell growth inhibition, a dose of 5 µM of SFN and 25 nM of DAC were chosen based on cell viability assays with more than 50% cells surviving from a single treatment of SFN and DAC respectively. Viable cell counts were calculated in the single and combination treatment of DAC and SFN compared to control (Figure 1B). There was 58% ± 4% and 56% ± 7% viable cells compared to control for the single treatment with DAC and SFN, respectively and only 33% ± 5% of viable cells in SFN and DAC combination treatment. The combination treatment induced significant growth inhibition compared to any single treatment ( *p* < 0.03, Student’s *t*-test).

### 3.2. SFN and DAC Single and Combination Treatment Result in Minimal Apoptosis 

Apoptosis analysis showed that most of the cells were noted as alive by negative stain for annexin V and DAPI in all treatments and control (Figure 1C,D). The percentage of viable cells not in apoptosis with DAC and SFN single treatments, was 99% ± 0.2% (*p* < 0.01) and 97% ± 1%( *p* < 0.01), respectively, compared to control. Combination treatment of DAC and SFN results in 95% ± 1% (*p* < 0.004) of viable cells compared to control. The percentage of viable cells not in apoptosis with combination treatments was slightly lower than any single treatment of SFN (*p* < 0.03) and DAC (*p* < 0.01).

### 3.3. SFN and DAC Single and Combination Treatment Result in No Cell Cycle Arrest

Cell cycle analysis indicated that all treated and control cells were in normal distributions for different cell cycles with G1 as dominant, followed by S phase and G2 phase, as shown in representative figures (Figure 1E). There was no significant difference between treatments in the G2/M phase (Figure 1F). 

### 3.4. SFN Induced Dysregulated Gene Transcription 

**Figure 2:**
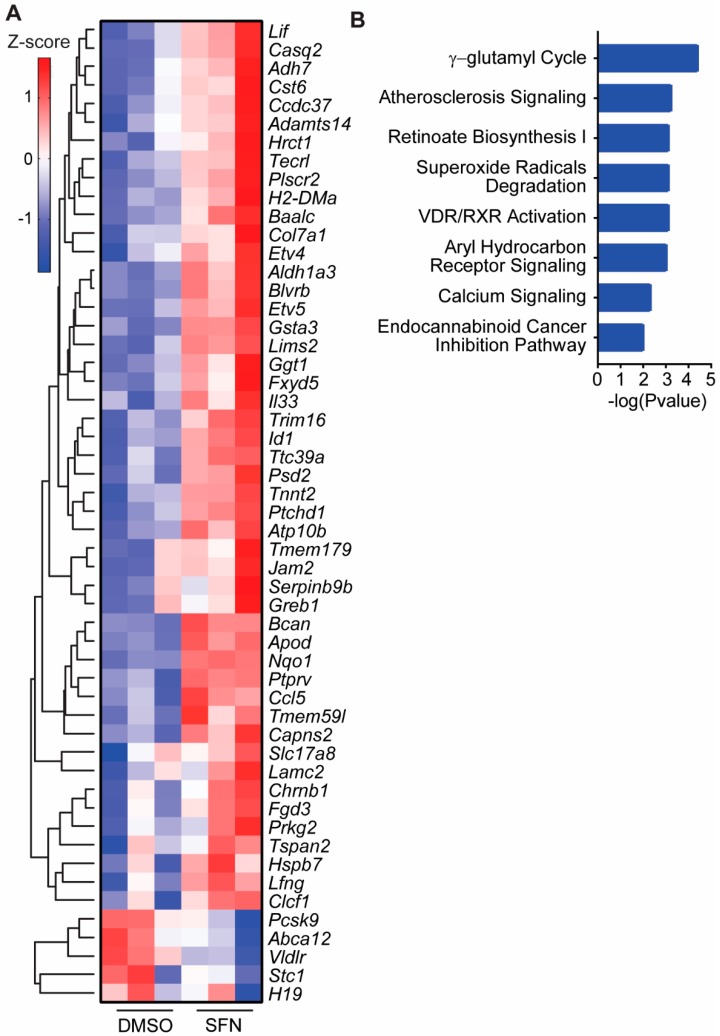
Differential gene expression induced by SFN single treatment and the related biological pathways. (**A**) Differentially expressed genes from SFN single treatment compared to control. Genes with greater than 2.5 fold changes (*p* < 0.001) were analyzed with unsupervised clustering (Z score shown in the color key). (**B**) Top canonical pathways from SFN single treatment. Genes greater than two-fold change relative to control were analyzed with Ingenuity Pathway Analysis (IPA) for their biological significance. The top eight pathways are shown here.

RNAseq data analysis revealed a differential gene expression profile by SFN single treatment compared to control. There were 126 genes with greater than 2-fold change compared to control. The data have been deposited in NCBI’s Gene Expression Omnibus [69]. The top genes with greater than 2.5-fold change (*p* < 0.001) are shown in the heatmap (Figure 2A). The biological roles of genes responding to SFN single treatment with greater than 2-fold change were analyzed with IPA. The top canonical pathways analysis, with a negative log p-value greater than 2, indicated many important biological pathways dysregulated in response to SFN single treatment (Figure 2B). 

### 3.5. SFN and DAC Uniquely Induced Dysregulated Gene Transcription 

**Figure 3:**
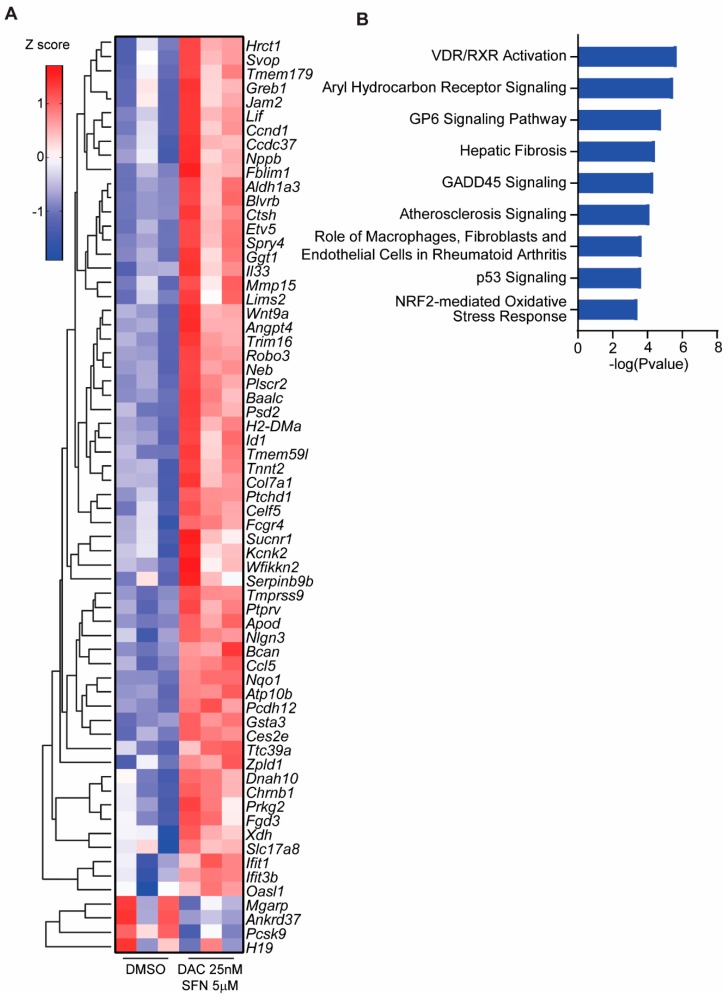
Differential gene expression induced by SFN and DAC combination treatment and the involved biological pathways. (**A**) Differentially expressed genes from the combination treatment of SFN and DAC compared to control treatments. Genes with greater than 3-fold change (*p* < 0.001) were analyzed with unsupervised clustering (Z score shown in the color key). (**B**) Top canonical pathways from the combination treatment of SFN and DAC. Genes greater than 2-fold change than the control with the combination treatment of SFN and DAC were analyzed with IPA for their biological significance. The top nine pathways are shown here.

DAC single treatment induced 19 genes to greater than 2-fold change compared to control (*p* < 0.05), and this number is too low for canonical pathway analysis by IPA. However, SFN and DAC combination treatment induced more genes than any single treatment. There were 261 genes with greater than a 2-fold change from the combination treatment of SFN and DAC compared to control (*p* < 0.05). The data from DAC single and SFN and DAC combination treatment have been deposited in NCBI’s Gene Expression Omnibus [69] as described above for SFN single treatment with the same accession number GSE12752. The top genes with greater than 3-fold change (*p* < 0.001) induced from SFN and DAC combination treatment are shown in the heatmap (Figure 3A). The biological roles of genes responded to SFN and DAC combination treatment compared to control with greater than 2-fold change were analyzed with IPA. The top canonical pathways analysis, with a negative log *p*-value greater than 3.5, showed many biological pathways involvement (Figure 3B). The role of vitamin D receptor/retinoid X receptor (VDR/RXR) activation and aryl hydrocarbon receptor signaling were listed as the top two canonical pathways from SFN and DAC combination treatment with a negative log *p*-value greater than 5.5. These two pathways were also detected in SFN single treatment with a negative log *p*-value approximately 3.0 (Figure 2B). 

### 3.6. Validation of Dysregulated Gene Transcription Induced by SFN and DAC Combination Treatment

**Figure 4:**
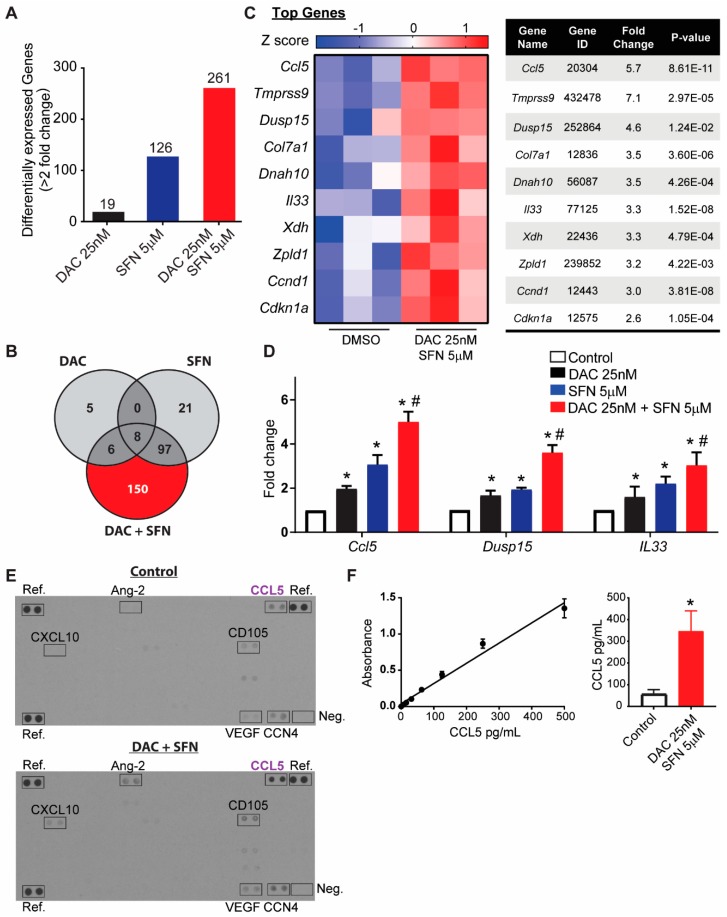
Validation of combination effects from SFN and DAC. (**A**) The number of differentially expressed genes induced by different treatments. (**B**) The number of unique genes responding to single and combination treatment. All genes were selected from greater than 2-fold change compared to control with *p* < 0.05. (**C**) A heatmap and list of top selected genes from SFN and DAC combination treatments (Z score shows in the color key). (**D**) Relative gene expression validation by rtPCR of Ccl5, Dusp15, and IL33 between treatments. * Significantly different from control, *p* < 0.05, # Single treatment is significantly different from combinational treatment, *p* < 0.05 (Student’s *t*-test). (**E**) Expression of cytokines detected by cytokine array. Ref indicates reference spots. Neg: negative control. Arrays were performed in duplicate. (**F**) Specific CCL5 ELISA further confirmed the increase in CCL 5 in DAC/SFN combination treatment. The left side indicated the standard curve of CCL5, ranged from 7.8 pg/mL to 500 pg/mL. The right side indicates the concentration of CCL5 in the supernatant is increased from 55pg/mL in control to 348 pg/mL in DAC/SFN combination treated group. All data were from two independent biological runs.

There were 261 genes with greater than 2-fold change (*p* < 0.05) of gene expression (either increased or decreased) with DAC plus SFN combination treatments compared to control. The number of genes with expression changes greater than 2-fold (*p* < 0.05), compared to control, from the single treatment were 19 and 126 genes for DAC and SFN, respectively (Figure 4A). Furthermore, there were 150 unique genes from combination treatment compared to control (Figure 4B). 

We selected genes for further validation from the SFN and DAC combination treatment with greater than 2-fold change compared to control. The preliminary selection criteria from RNA-seq data were genes with the highest differential expression compared to control or involved in multiple top biological pathways (Figure 4C). Three genes, CCL5, DUSP15, and IL33, were validated by reverse transcription PCR (RT-PCR) (Figure 4D). These genes were selected for validation on the criteria that they showed differential expression between single treatment and control as well as between combination treatment and single treatment. CCL5 increased 2 ± 0.1 (*p* < 2.4 × 10^−6^) and 3 ± 0.2 (*p* < 1.5 × 10^−5^) times with single treatment of DAC and SFN, respectively, and increased 5 ± 0.2 (*p* < 1.3 × 10^−7^, Student’s *t*-test) with combination treatment compared to control. DUSP15 increased 1.7 ± 0.2 (*p* < 6.2 × 10^−3^) and 1.9 ± 0.1 (*p* < 8.7 × 10^−6^) times with single treatment of DAC and SFN, respectively, and increased 3.6 ± 0.3 (*p* < 1.7 × 10^−5^, Student’s *t*-test) with combination treatment compared to control. IL33 increased 1.6± 0.3 (*p* < 4.2 × 10^−2^) and 2.2 ± 0.2 (*p* < 2.0 × 10^−4^) times with single treatment of DAC and SFN, respectively, and increased 3.0 ± 0.3 (*p* < 2.1 × 10^−4^, Student’s *t*-test) with combination treatment compared to control. 

The level of secreted cytokines CCL5 and IL33, as well as other 111 cytokines, were measured in the supernatant using a mouse XL cytokines array (Figure 4E). Out of the 111 mouse cytokines probes on the membrane, CCL5 was detected with greater than two times increased signal in combination treatment compared to the control group. IL33 was not present at detectable levels. Other cytokines, such as CXCL10 (Gene ID 15945), angiopoietin-2 (Gene ID 11601), CD105 (Gene ID 13805), VEGF (Gene ID 22339), and CCN4 (Gene ID 22402), were detected with an increased level of expression in combination treatment than control.

Specific CCL5 ELISA further confirmed the increase in CCL 5 in DAC/SFN combination treatment, as indicated in Figure 4F. The level of CCL5 in control is about 55 +/− 22.3 pg/mL and is increased to 348 +/− 92.2 pg/mL in SFN/DAC combination treatment from two independent biological runs. 

### 3.7. Analysis of Histone Epigenetic Post-Translational Modifications (PTMs) 

Post-translational modifications were identified and subsequently quantified on histones H3 and H4. EPZ treatment is known to decrease H3K27me3, and was consequently used as a positive control. We treated cells with EPZ6438 for 48 hrs along with SFN and DAC combination treatments as described in the methods section. Histone PTMs were analyzed using lysine derivatization and a bottom-up proteomic workflow. Aside from positive control, no significant differences in histone PTMs were detected (Supplementary Figure S1). 

## 4. Discussion

For the current study, we explored the possibility of controlling melanoma cell growth by combining the antioxidant SFN and the epigenetic drug DAC. The rationale behind this work was to control the level of ROS while altering the epigenetic status with a relatively low dose of each drug. The aim is to lay the first step for our long term goal in using a dietary dose of an antioxidant to help epigenetic drugs in controlling melanoma. Therefore, we aimed to use a low dose of each drug to allow the future application of a dietary dose of SFN and a low dose of DAC to reduce side effects. We chose 5 µM of SFN and 25 nM of DAC, which are equal to or lower than half of the respective IC50 from our test (Figure 1A). These doses of the drugs induce significant growth inhibition with combination treatment compared to control and either single treatment (Figure 1B). We did not find apoptosis or cell cycle arrest in any treatments (Figure 1C–F). This finding is different from other studies using a higher dose of each drug (6–25 µM of SFN [70,71] and 200 nM–0.5 µM of DAC [72,73]). These higher-dose studies all demonstrate apoptosis and cell arrest effects. At the low doses used in this study, the two drugs induced different mechanisms as compared to studies using relatively high doses of SFN or DAC. Our findings suggest that the growth inhibition may be involved in mechanisms other than apoptosis and cell cycle arrest. A combination treatment of low-dose SFN and DAC reduced the cell growth without initiating cell cycle arrest or apoptosis. These data indicate that attenuating ROS with the antioxidant SFN may enhance the utility of the epigenetic drug DAC in controlling cell growth, with less impact on the host. 

We investigated the impact of this drug combination at the transcriptional level by RNAseq (Figure 2 and Figure 3). There was a significant increase in the total number of genes with greater than 2-fold (*p* < 0.05) expression change in cells that received the combination treatment as compared to those that received either single treatment and as compared to control (Figure 4A). The absolute number of genes altered by 25 nM of DAC is very low at 19, and those altered by SFN alone is higher at 126. This may be attributed to the low dose treatment with limited impact. Interestingly, the number of altered genes increased to 261 when SFN and DAC combination treatment was applied. The top differentially-expressed genes and canonical pathways showed different distributions between single SFN and combination SFN and DAC treatment (Figure 2 and Figure 3). VDR/RXR activation and aryl hydrocarbon receptor signaling (AhR receptor) are two top-listed pathways from combination treatments. Both pathways are known to be associated with UV exposure [5,15,74,75,76]. We validated select genes involved in more than one canonical pathway or listed as top differentially-expressed genes (Figure 4C). The transcription level of the three genes (CCL5, IL33, and DUSP15) were significantly higher in the combination treatment than either of the single treatments (Figure 4D). Two (CCL5 and IL33) of the three genes are secreted proteins. We further validated secreted proteins with cytokine arrays and ELISA on CCL5 (Figure 4E,F). CCL5 was validated to have increased levels, both in transcription level and detected extracellularly after combination treatment as compared to control. CCL5 is also known as RANTES (regulated on activation, normal T cell expressed and secreted). It is one of the cytokines which functions as a chemoattractant for natural killer (NK) cells [77], which do not efficiently infiltrate solid tumors such as melanoma [78]. CCL5 is the main factor in inhibiting melanoma growth by bringing NK cells to the tumor site, while autophagy is suppressed [79]. Activated NK cells could stimulate the immune checkpoint programmed cell death protein 1 (PD-1) [80] and cytotoxic T lymphocytes (CTL)-associated antigen 4 (CTLA4) [81] to deliver immunoregulatory effects. Increased expression of CCL5 involves the phosphorylation of the MAPK8/JNK-JUN/c-Jun signaling pathway, which is initiated by decreased expression of protein phosphatase 2 A (PP2A), while autophagy is suppressed [82,83]. Clinically, a high level of CCL5 is positively associated with the NK cell marker NKp46 as well as with melanoma patients’ survival [79,84]. 

We also investigated whether low dose treatments of SFN and DAC have an impact on histone PTMs. There was no differential PTMs detected when control and combination of SNF and DAC treated cells were analyzed (Supplementary Figure S1). This suggests that under the conditions of our treatments, the differential gene expression and cell inhibition may not be associated with histone epigenetic reprogramming, but rather the direct effects of SFN and DAC.

In summary, our data suggest that attenuating ROS through the use of the antioxidant SFN can help the epigenetic drug DAC control cell growth. This control is not via direct cell killing with apoptosis, cell cycle arrest or histone modifications, but, more directly, by changing gene transcription and cytokine production, which may increase the immune defense system by recruiting natural killer cells. 

## 5. Conclusions

Melanoma patients not only have high oxidative stress [23], but also have a high frequency of recurrence of the disease [85,86]. It is apparent that melanoma patients are susceptible to daily UV- and environmental exposure-induced ROS [5]. Managing the level of ROS via natural antioxidants has demonstrated beneficial effects in controlling melanoma [5], but does not eliminate the tumor. Our study aimed to attenuate ROS by a low dose of the antioxidant SFN and allow the epigenetic drug DAC to control melanoma at a lower dose. The current study clearly demonstrates that SFN could have combinational effects with the commonly used, FDA-approved demethylation agent DAC in significantly inhibiting melanoma cell growth. The next goal is to apply our findings to animal studies. The long term goal is for the clinical application of controlling melanoma with a dietary dose of SFN and target drugs (e.g., epigenetic and immunotherapeutic drugs) at lower doses that may have fewer side effects for patients. 

## Figures and Tables

**Table 1:** Primers.

Ccl5-Forward	ACCATATGGCTCGGACACCA
Ccl5-Reverse	TCTCTGGGTTGGCACACACTT
IL33-Forward	GGGGCTCACTGCAGGAAAGT
IL33-Reverse	ATTTTGCAAGGCGGGACCAG
Dusp15-Forward	TATCCACGAATCACCCCA
Dusp15-Reverse	AAGCAGTGCACAAGGCA
UBC-forward	GCCCAGTGTTACCACCAAGAGCC
UBC-Reverse	CCCATCACACCCAAGAACAGTT

Ccl5: (C-C motif) ligand 5 (Gene ID:20304); IL33: interleukin 33 (Gene ID:77125); Dusp15: dual specificity phosphatase-like 15 (Gene ID:252864); UBC: Ubiquitin C (Gene ID: 22190).

**Table 2:** List of mouse histones used for analysis.

Mouse Histones					
H10	H1FOO	H2A2B	H2AX	H2B1H	H2B3B
H11	H1T	H2A2C	H2AY	H2B1K	H31
H12	H2A1	H2A3	H2AZ	H2B1M	H32
H13	H2A1F	H2AB1	H2B1A	H2B1P	H33
H14	H2A1H	H2AJ	H2B1B	H2B2B	H3C
H15	H2A1K	H2AV	H2B1C	H2B2E	H4
H1FNT	H2A2A	H2AW	H2B1F	H2B3A	

## Supplementary Materials

The following are available online at https://www.mdpi.com/2305-6320/6/3/71/s1, Figure S1: Histone post-translational modifications on Histone H3 (A) and H4 (B) were detected upon EPZ6438 treatment as well as SFN and DAC combination treatment. As anticipated for the positive control, H3K27me3 was significantly lower following EPZ treatment.
