# Supplementary material for: Effect of Sulforaphane and 5-Aza-2’-Deoxycytidine on Melanoma Cell Growth
Source: Medicines (Basel). 2019 Jun 27;6(3):71. doi: 10.3390/medicines6030071 (PMC6789461; doi:10.3390/medicines6030071)
Supplement: Supplementary file 1 [file medicines-06-00071-s001.pdf]

Supplementary Materials: Effect of Sulforaphane and 5-Aza-2'-Deoxycytidine on Melanoma Cell Growth

Tung-chin Chiang, Brian Koss, L. Joseph Su, Charity L. Washam, Stephanie D. Byrum, Aaron Storey and Alan J. Tackett

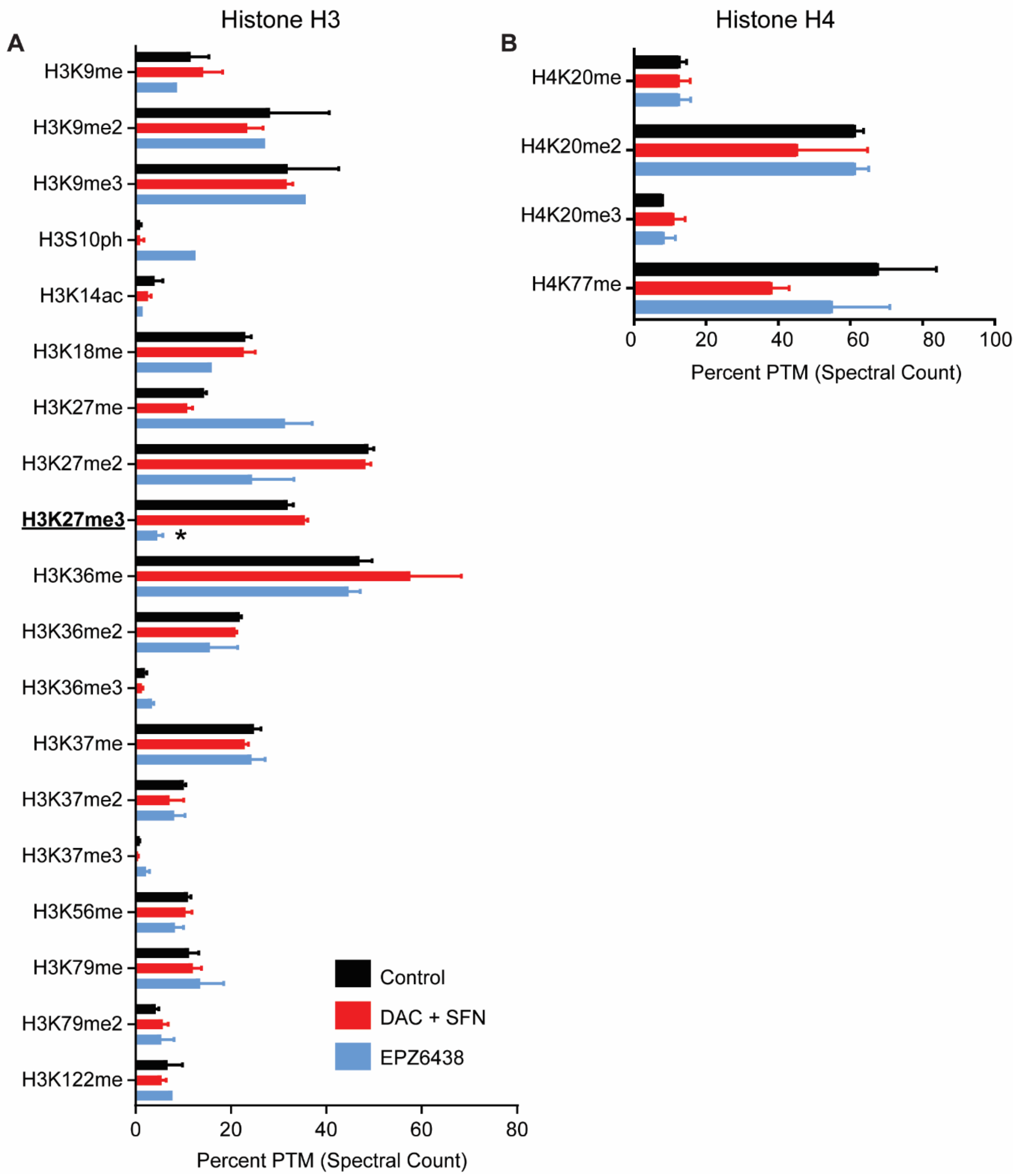

Figure S1. Histone post-translational modifications on Histone H3 (**A**) and H4 (**B**), were detected upon EPZ6438 treatment as well as SFN and DAC combination treatment. As anticipated for the positive control, H3K27me3 was significantly lower following EPZ treatment.
